# Nontraumatic warfarin-related intrapulmonary hemorrhage presenting as a lung mass

**DOI:** 10.1186/s40792-020-00830-z

**Published:** 2020-04-10

**Authors:** Hiroyuki Tsuchida, Ryo Fujikawa, Hidenori Nakamura, Toru Nakamura

**Affiliations:** 1 Department of General Thoracic Surgery, Seirei Hamamatsu General Hospital, 2-12-12, Sumiyoshi, Naka-ku, Hamamatsu, Shizuoka, 430-8558 Japan; 2 Department of Respiratory Medicine, Seirei Hamamatsu General Hospital, 2-12-12, Sumiyoshi, Naka-ku, Hamamatsu, Shizuoka, 430-8558 Japan

**Keywords:** Warfarin, Intrapulmonary hemorrhage, Anticoagulation, Bleeding events

## Abstract

**Background:**

Although bleeding events are the major concern when using oral anticoagulants, intrathoracic hemorrhages due to warfarin are rare. Most cases in the literature have been related to trauma and have manifested as a hemothorax. Here we report a case of a nontraumatic hemorrhage within a pre-existing pulmonary cyst that presented as a lung mass during warfarin therapy.

**Case presentation:**

A 75-year-old asymptomatic woman presented with a 10-cm-diameter mass on chest radiography that was not evident 6 months prior. She had been taking warfarin for paroxysmal atrial fibrillation and a transient ischemic attack. There was no history of chest trauma, warfarin overdosing, or any suspected interactions with other drugs such as nonsteroidal anti-inflammatory drugs or antibiotics. The prothrombin time/international normalized ratio(PT-INR) was prolonged at 4.73 and her hemoglobin level was 8.7 g/dl. Chest computed tomography(CT)revealed an air-fluid mass adjacent to the right upper and middle lobes with a pleural effusion. A CT scan obtained 15 years prior revealed a cyst at the corresponding site and the mass was diagnosed as a warfarin-related hemorrhage within the pre-existing pulmonary cyst. We performed a surgical resection of the cyst to prevent any worsening hemorrhage and subsequent infection. The postoperative course was uneventful and the patient was discharged on the 3rd postoperative day.

**Conclusion:**

A warfarin-related thoracic hemorrhage, other than a hemothorax, could manifest as a pulmonary mass on radiography in patients with pre-existing pulmonary cysts. History taking especially of any anticoagulant medications and a precise assessment of the past images are crucial for a correct diagnosis. Once the intrapulmonary cystic hemorrhage becomes evident, prompt withdrawal with a reversal of warfarin and surgical resection are required to prevent a worsening hemorrhage and subsequent infection.

## Background

Although bleeding events are the major concerns of oral anticoagulant use, intrathoracic hemorrhages due to warfarin are rare with a frequency of 3% [1]. Most cases in the literature have been related to trauma and have manifested as a hemothorax. To the best of our knowledge, there have been no reports of intrapulmonary hemorrhages associated with warfarin. Here we report a case of a nontraumatic hemorrhage within a pre-existing pulmonary cyst presenting as a lung mass during warfarin therapy.

## Case presentation

**Fig. 1:**
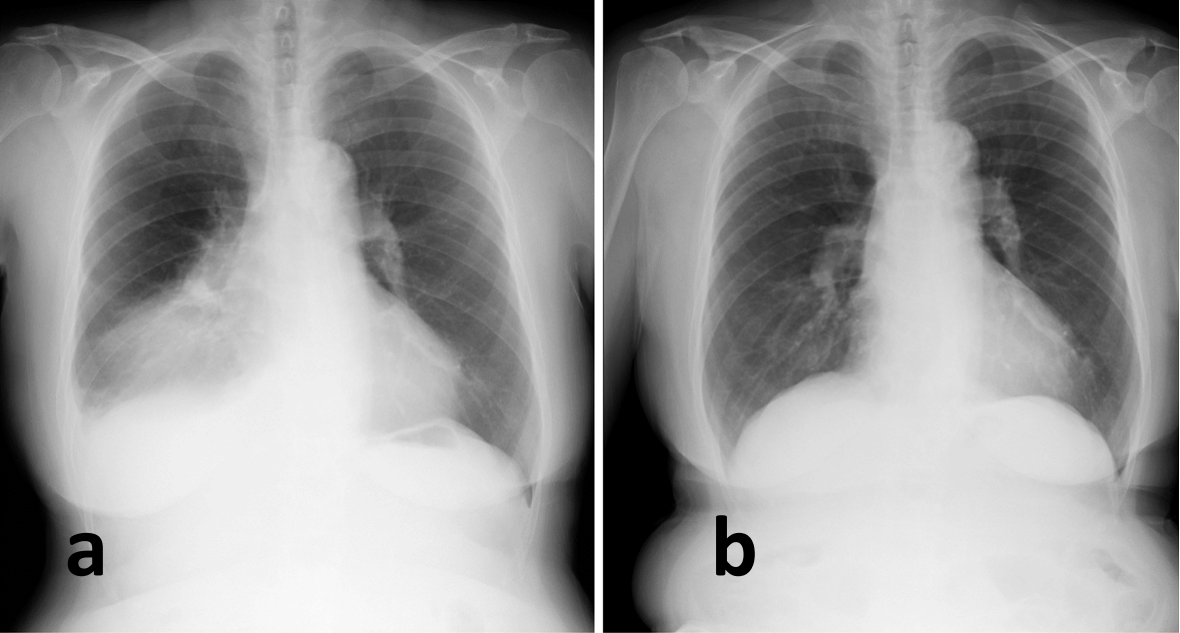
A chest radiograph on admission revealed a mass (**a**) which was not evident 6 months prior (**b**)

**Fig. 2:**
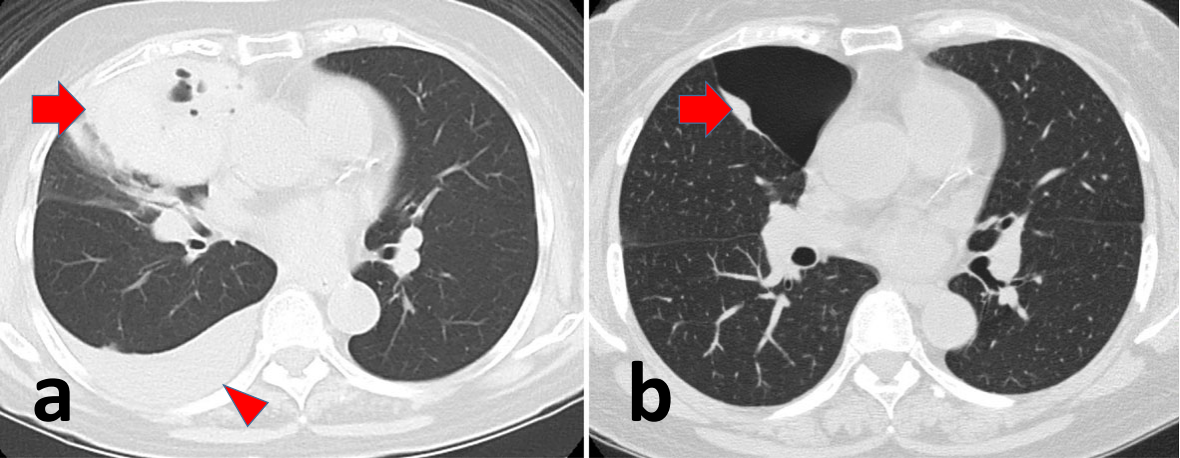
**a** A chest CT on admission revealed an air-fluid mass (arrow) adjacent to the right upper and middle lobe with a pleural effusion (arrowhead). **b** A CT scan obtained 15 years prior revealed a cyst(arrow)at the corresponding site

A 75-year-old asymptomatic woman presented with a 10-cm-diameter mass on her chest radiograph, which was not evident 6 months prior (Fig. 1). She had been taking warfarin for paroxysmal atrial fibrillation and a transient ischemic attack for 6 years. There was no history of chest trauma, warfarin overdosing, or suspected interactions with other drugs such as nonsteroidal anti-inflammatory drugs or antibiotics. She had no fever, leukocytosis, or elevated C-reactive protein. The prothrombin time/international normalized ratio (PT-INR) was prolonged at 4.73 and her hemoglobin level was 8.7 g/dl. Chest computed tomography(CT)revealed an air-fluid mass adjacent to the right upper and middle lobes with a pleural effusion (Fig. 2a). A CT scan obtained 15 years prior revealed a cyst at the corresponding site (Fig. 2b), and the mass was diagnosed as a warfarin-related hemorrhage within the pre-existing pulmonary cyst. Immediately after the withdrawal of the warfarin and administration of vitamin K, the PT-INR normalized to 0.96 without any further progression of the anemia or remarkable change in the mass and effusion. A surgical resection of the cyst to prevent a worsening hemorrhage and subsequent infection was planned.

**Fig. 3:**
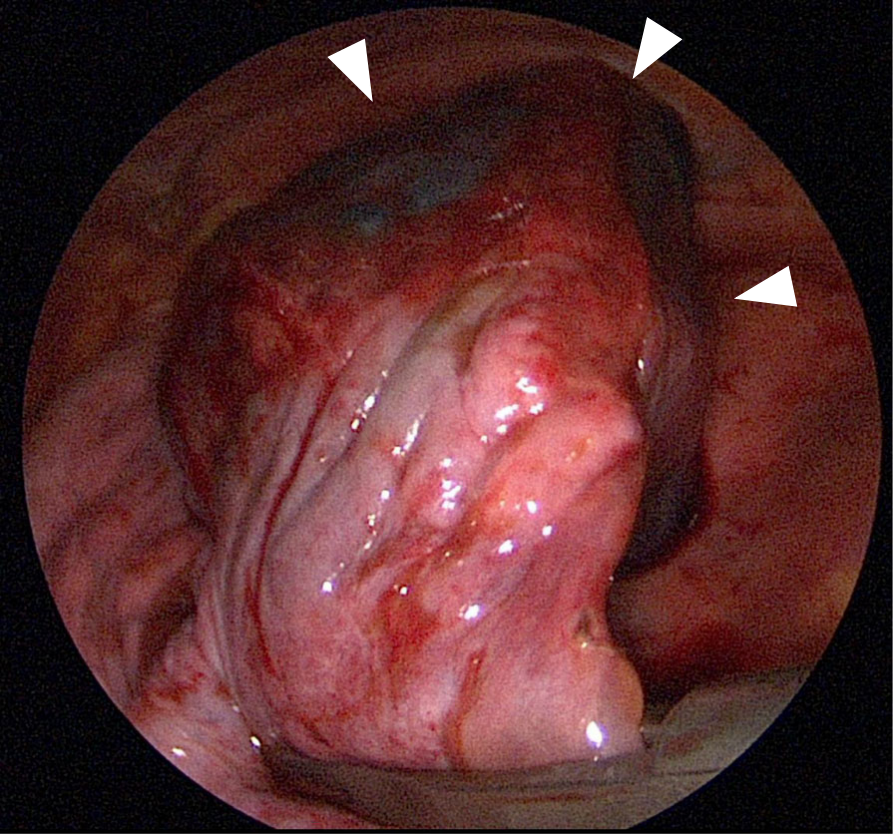
A thoracoscopic view revealed a pulmonary cyst filled with a clot and bloody effusion (arrowheads)

Thoracoscopic view revealed the pulmonary cyst filled with a clot and 650 ml of bloody effusion (Fig. 3). We performed wedge resection of the affected mass mainly arisen from the middle lobe. No intraoperative frozen section examination was performed. There were no other obvious bleeding points. The operation time was 102 min and the blood loss was 5 ml. The pathological findings revealed an emphysematous bulla filled with a blood clot with an alveolar hemorrhage. The cause of the bleeding remained uncertain because there were no neoplastic lesions with any specific blood vessels involved in the resected specimen. The postoperative course was uneventful and the patient was discharged on the 3rd postoperative day. A direct oral anticoagulant (DOAC) was administered thereafter. She currently remains disease-free at 2 months after the operation.

## Comments

Our case exhibited two important issues. The first was that an intrapulmonary hemorrhage, other than a hemothorax, could occur as an anticoagulant-related bleeding event. Warfarin is one of the leading causes of drug-related adverse events [1–3], and its effect is influenced by several factors such as a genetic variation and drug interactions with a narrow therapeutic range by which bleeding events could occur. It could involve gastrointestinal, urinary tract, and intracranial bleeding, whereas thoracic hemorrhages are rare, with only a few cases having been reported to date [2, 4–7]. Most cases in the literature manifested as a hemothorax associated with trauma, and to the best of our knowledge, this is the first report of a nontraumatic intrapulmonary hemorrhage during warfarin therapy. The etiology of the sudden hemorrhage in our case was unclear and without a history of trauma or the concomitant use of any suspected drugs, which may have interacted with warfarin. An additional precise interview of the patient failed to identify any interpretable contributing factors such as a relevant diet.

The second point was that a hemorrhage inside a pulmonary cyst could manifest as a mass, without a diffuse opacity, in cases with a typical alveolar hemorrhage on radiography. While a neoplastic lesion was raised as a differential diagnosis during the initial workup, warfarin use with a prolonged PT-INR and the pulmonary cyst visualized on the CT scan 15 years prior made a diagnosis of a drug-related hemorrhage in the present case. We thoracic surgeons should be aware of the possibility that an intrapulmonary hemorrhage could manifest with such an atypical radiological finding in patients with pre-existing pulmonary cysts.

Evacuation of a bloody effusion is essential for a thoracic hemorrhage mainly because the retained clot could cause subsequent empyema [8, 9]. While an adequate tube thoracostomy might be sufficiently effective in most cases, a surgical debridement was required in the present case because the intrapulmonary hemorrhage was more significant than a hemothorax. With a prompt reversal of warfarin, the affected area was successfully resected and there have been no bleeding events even after the administration of DOACs to date.

## Conclusions

Warfarin-related thoracic hemorrhages other than a hemothorax could manifest as a pulmonary mass on radiography in patients with a pre-existing pulmonary cyst. History taking especially for anticoagulant medications and a precise assessment of the past images are crucial for a correct diagnosis. Once an intrapulmonary cystic hemorrhage becomes evident, prompt withdrawal and reversal of warfarin and a surgical resection are required to prevent a worsening hemorrhage and subsequent infection.

## Data Availability

Not applicable.

## Abbreviations

PT-INR: Prothrombin time/international normalized ratio
CT: Computed tomography
